# Clampless and sutureless laparoscopic partial nephrectomy using monopolar coagulation with or without *N*-butyl-2-cyanoacrylate

**DOI:** 10.1186/s12957-019-1614-8

**Published:** 2019-04-17

**Authors:** Feng Zhang, Shuang Gao, Xiao-Nan Chen, Bin Wu

**Affiliations:** 1 Department of Urology, Shengjing Hospital of China Medical University, 36 Sanhao Street, Heping District, Shenyang, 110004 China; 2 Department of pathology, People’s Hospital of Liaoning Province, 33 Wenyi Road, Shenhe District, Shenyang, 110016 China

**Keywords:** Hemostasis, Laparoscopy, Nephrectomy, Organ-sparing treatments

## Abstract

**Objective:**

To describe a novel technique for clampless and sutureless laparoscopic partial nephrectomy (LPN) using monopolar coagulation with or without *N*-butyl-2-cyanoacrylate (NBCA).

**Methods:**

From February 2015 to October 2018, we performed clampless and sutureless LPN using monopolar coagulation with or without NBCA on 142 patients. The tumors were resected with cold scissor. The tumor beds were repeatedly coagulated with a monopolar hook in spray and fulgurate modes. NBCA was sprayed when bleeding was observed after coagulation in 98 patients. We compared outcomes in the NBCA and non-NBCA groups.

**Results:**

Mean patient age was 55 years (range 20–86). Mean tumor size was 3.2 cm (range 1.0–10.6). Mean RENAL nephrometry score was 5 (range 4–8). Mean operative time was 120 min (range 40–200). Mean estimated blood loss was 100 ml (range 10–500). Mean eGFR changes were 2.3 ml/min. Two patients had positive surgical margins. Three patients received blood transfusions. No patients had urine leakage. Patients receiving NBCA had larger tumors (3.0 vs 2.0 cm, *p* < 0.001), higher RENAL nephrometry scores (5.59 vs 4.47, *p* = 0.004), and higher E item scores (*p* = 0.009).

**Conclusions:**

Use of monopolar coagulation with NBCA in clampless and sutureless LPN for renal tumors with low RENAL nephrometry scores is safe and effective. For patients with exophytic renal tumors less than 2 cm, NBCA is not necessary.

## Introduction

The number of incidentally discovered renal masses has increased in the era of ultrasound (US) and by computed tomography (CT). Based on oncological and functional outcomes, localized renal tumors had been better managed by partial nephrectomy (PN) rather than by radical nephrectomy (RN) [1]. Laparoscopic partial nephrectomy (LPN) gave equivalent oncologic and functional outcomes to those of open PN [2]. Nevertheless, the steep learning curve and technical demands of LPN make this technique challenging for adoption as a new procedure. The ultimate goal is the achievement of the PN “trifecta”: negative surgical margins, functional preservation, and complication-free recovery [3].

Classical LPN techniques call for clamping of renal vessels to occlude the blood supply to the kidney, creating a bloodless field. Tumors were excised along with margins of normal parenchyma. After tumor excision, transected intrarenal blood vessels and the collecting system were repaired with sutures to ensure hemostasis and water-tight closure. The incision in the kidney was closed by suturing [4]. To minimize ischemic renal injury, the recommended clamp time was less than 30 min [5].

Increasing concern has been placed on the issue of renal function preservation following PN, particularly with respect to reduction of warm ischemia time. The precise segmental renal artery clamping technique [6], the off-clamp technique [7], and the early unclamping technique [8] have been used to accomplish this.

More recently, reconstruction of the renal parenchyma has been emphasized. L’Esperance et al. [9] found that deep corticomedullary sutures placed during PN affected renal function by closing off important arteries. Bahler and Sundaram [10] found that the reconstruction of the renal remnant had the most substantial impact on renal function. A single-layer running suture, particularly using barbed thread, shortened operating and ischemia times [11]. Porpiglia et al. [12] described a suturing method that minimized the ischemic effect on the renal parenchyma. Other surgeons invented some techniques to achieve a sutureless procedure [13–15].

In the present study, we present our experiences with clampless and sutureless LPN using monopolar coagulation. *N*-butyl-2-cyanoacrylate (NBCA) was selectively used in various scenarios to control bleeding. We evaluated the efficacy of the technique in the initial cases and reported perioperative outcomes.

## Patients and methods

A total of 142 patients underwent clampless and sutureless LPN using monopolar coagulation with or without *N*-butyl-2-cyanoacrylate (NBCA) from February 2015 to October 2018 at the Shengjing Hospital of China Medical University in Shenyang, China. The study was approved by the Shengjing Hospital’s ethics committee (No.2018PS012J). All patients were informed of the potential complications and risks of the surgeries. The RENAL nephrometry scores were assessed by a single doctor based on perioperative CT scans. All the surgeries were performed by Bin Wu. The inclusion criteria were maximum tumor diameter within renal parenchyma was less than 4 cm, 1–2 points on item E of the RENAL score, and 1–2 points on item N of the RENAL score.

The transperitoneal approach was performed in 122 patients, and the retroperitoneal approach was performed in 20 patients with tumors located at the posterior and middle sides of the kidney, respectively. Changes in eGFR were evaluated by the difference in serum creatinine preoperatively and 6 months postoperatively. Individual renal function was not evaluated. No cooling procedures were carried out. Two milligrams of *N*-butyl-2-cyanoacrylate (NBCA, Compont®, Beijing, China) was sprayed on the tumor bed in 98 patients.

Patient demographics (gender, age); disease characteristics (tumor side and size, RENAL score); intraoperative data (surgical approach, duration of surgery, estimated blood loss); and postoperative data (comorbidity, positive surgical margins) were recorded.

### Technique

**Fig. 1:**
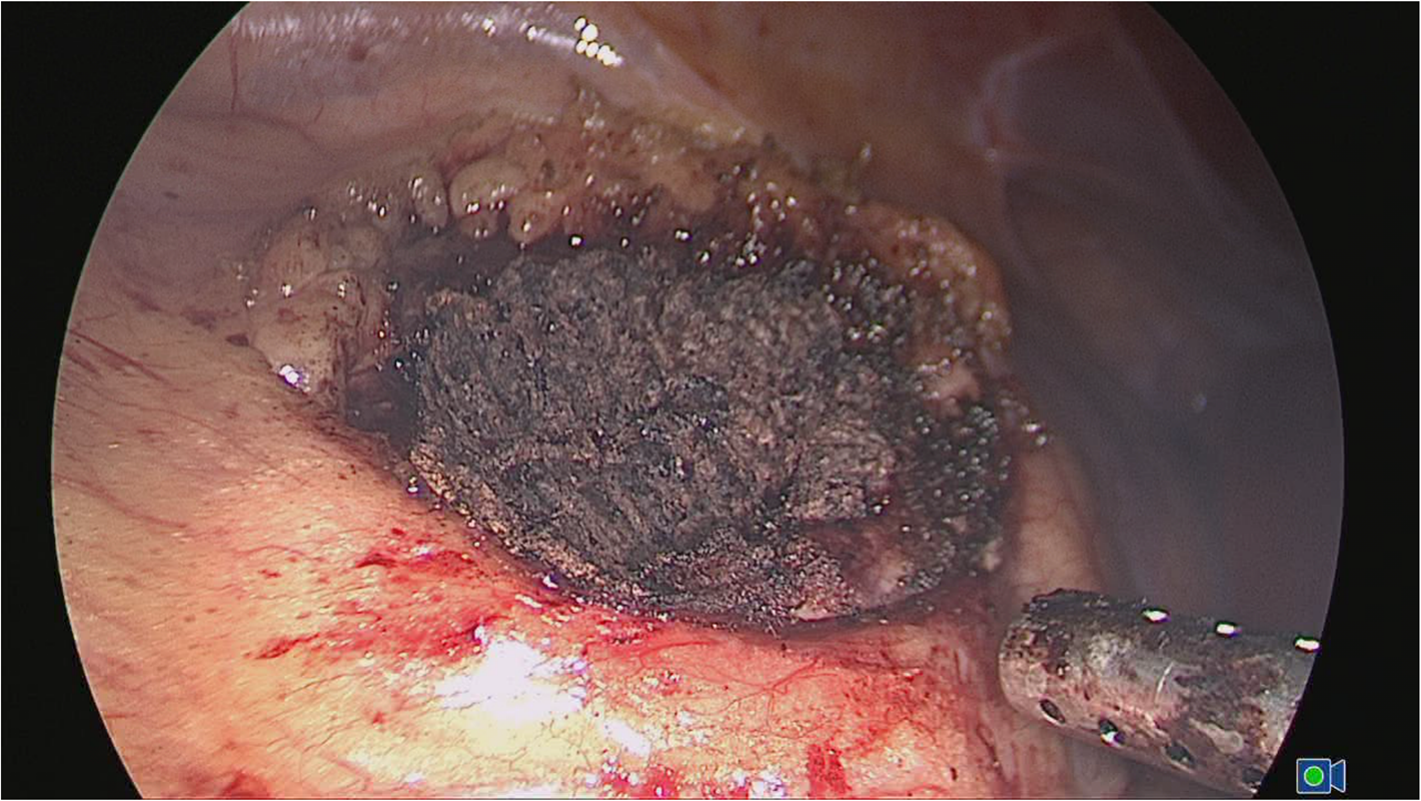
Repeated coagulation to form a helmet-like eschar helped maintain hemostasis

**Fig. 2:**
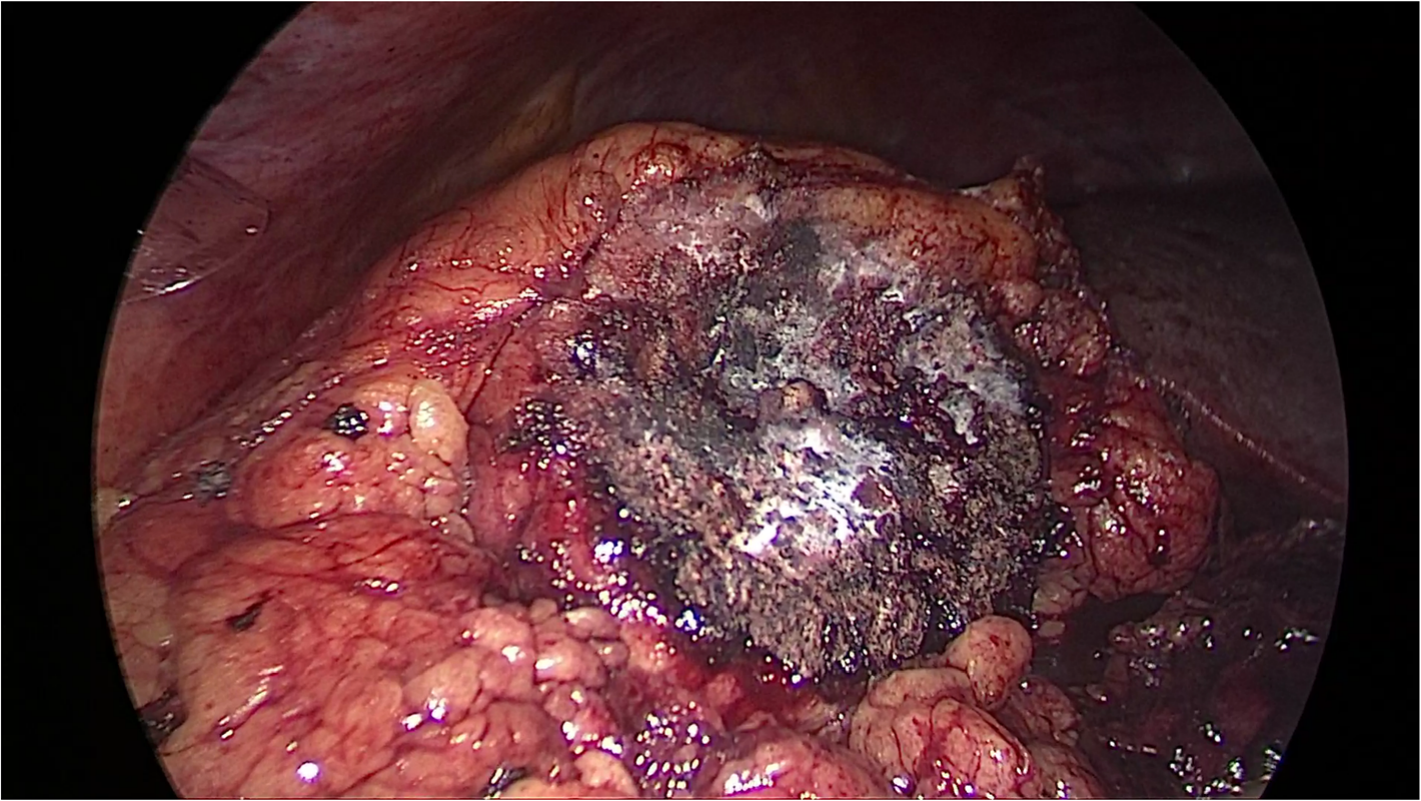
To achieve satisfactory hemostasis, NBCA was sprayed on the surface of the tumor bed. The NBCA solidified in less than 1 min

We used the transperitoneal or retroperitoneal approach with a three-trocar technique and the patient in the lateral decubitus position. Once entering the retroperitoneal space, we opened the Gerota’s fascia and perirenal fat to locate the tumor. The fat tissue overlying the tumor was preserved when feasible. Renal hilar was isolated when the tumor diameter was larger than 3 cm to prevent catastrophic hemorrhage. The renal parenchyma around the tumor was marked using monopolar coagulation. The tumors were excised with cold scissors and bluntly dissected and enucleated with a vacuum-assisted suction aspirator outside the pseudocapsule. It was important to avoid thermally cutting in order to keep the visual field clear and to ensure the capsule of the tumor remained intact. When hemorrhaging blood vessels were visualized, the monopolar hook was used in spray mode for coagulation. Once tumor excision was completed, repeated monopolar coagulation was carried out on the tumor bed in spray mode (100 W) and fulgurate mode (60 W) by a monopolar hook (Fig. 1). When bleeding was still observed, NBCA was sprayed on the tumor bed. NBCA solidified within 1 min on the traumatized surface and formed a helmet-like eschar to ensure hemostasis (Fig. 2). A drainage tube was placed near the tumor bed. The excised tumor was removed through a laparoscopic retrieval bag.

### Statistical analysis

Data were analyzed using SPSS 22.0 for Windows (SPSS Inc., Chicago, IL, USA). The means of two continuous normally distributed variables were compared using the independent samples Student’s test. The Mann-Whitney *U* test was used to compare two continuous non-normally distributed variables. The chi-squared and Fisher’s exact tests were used for comparison of categorical variables. A *p* value of less than 0.05 was considered statistically significant.

## Results

The characteristics and the surgical outcomes of the 142 patients are listed in Table 1. The mean age was 55 years (range 20–86). Gender distribution was 93 males and 49 females. The mean size of tumors was 3.2 cm (range 1.0–10.6). The mean RENAL nephrometry score was 5 (4–8). Histopathological findings revealed 103 renal cell carcinomas, 33 angiomyolipomas, and five oncocytomas. Mean operative time was 120 min (range 40–200). Mean estimated blood loss was 100 ml (range 10–500). Mean change in eGFR was 2.3 ml/min after 6 months follow-up. Three patients (RENAL score 7, 8, and 8) received blood transfusions (Clavien grade 2) for hemorrhage during or after surgery. Two patients had positive surgical margins. No urinary leakage was reported. No surgeries were converted to open.

**Table 1:** Characteristics and perioperative parameters

Characteristics and perioperative parameters	
No. of patients	142
Mean age (years)	55 (20–86)
Sex (male/female)	93 (65.5%)/49 (34.5%)
tumor sites (right/left)	79 (55.6%)/63 (44.4%)
Mean tumor size (range)	3.2 (1.0–10.6)
RENAL nephrometry score mean (range)	5 (4–8)
Low 4–6 (%)	114 (80.2%)
Medium 7–9 (%) 46 (30.2%)	28 (19.8%)
NBCA used (yes/no)	98 (69%)/44 (31%)
Mean operative time, min (range)	120 (40–200)
Mean estimated blood loss, ml (range)	100 (10–500)
Mean change in eGFR (ml/min)	2.3
Blood transfusion	3
conversion to open (*n*)	0
Positive surgical margin (*n*)	2
Histopathology	
Renal cell carcinoma	103
Angiomyolipoma	34
Oncocytoma	5

A total of 98 patients received NBCA intraoperatively, and 44 did not. The details are shown in Table 2. Patients receiving NBCA had larger tumors (3.0 vs 2.0 cm, *p* < 0.001), higher RENAL nephrometry scores (5.59 vs 4.47, *p* = 0.004), higher E item scores (*p* = 0.009), than did patients not receiving NBCA.

## Discussion

The majority of renal tumors were diagnosed at clinical stage T1 and were amenable to PN [16]. PN has been increasingly utilized to treat T2 renal tumors, and Bertolo et al. reported that PN in the setting of selected cT2 renal masses can be safely performed with acceptable outcomes [17]. However, the technique of LPN is much more difficulty than that of OPN, especially the suturing techniques in laparoscopy, and complications occurred more frequently [18].

Warm ischemia time (WIT), resected healthy margins, and reconstructive injury (renorrhaphy) all impacted renal remnant function [19]. The importance of WIT has been emphasized in the published PN literatures [20]. A WIT “cutoff point” of 30 min has been conventionally accepted as the safe limit for PN [5]. However, Thompson et al. [21] published a study entitled “Every minute counts”, in which they found that a decrease of every minute in WIT was beneficial in terms of renal function preservation. Gill et al. [7] declared every effort to minimize or eliminate ischemia during PN would be a welcomed step forward. In our study, all patients underwent zero-ischemia LPN, and it proved to be an excellent method for protecting renal function.

The acceptable healthy margin during PN resection has historically been considered to be 0.5–1cm. Sutherland et al. [22] proposed that 0.2cm was a safe margin. More recently, some studies claimed that simple tumor enucleation had similar oncologic outcomes to those of standard PN and RN [23, 24]. We used the enucleation method to excise the tumor, with combination of sharpness and blunt separation. Two (1.4%) positive surgical margins were found in our cases, a similar rate to the one reported by Minervini et al. [25].

The suture needle itself has been occasionally reported to transect or puncture arteries, leading to renal artery pseudoaneurysms [26]. More recently, Tanaka et al. [27] reported renal artery pseudoaneurysms were absent when they used their clampless and sutureless technique. Complications may be reduced if bleeding control could be ensured without renorrhaphy. Simone et al. [14] performed “zero ischemia” sutureless LPN with Liga SureTM for renal tumors with low nephrectomy scores on 101 patients. Hemostasis was achieved with coagulation and biological hemostatic agents. Ota et al. [15] performed PN using SOFT coagulation without renorrhaphy on 39 patients. Li et al. [13] reported LPN in 31 patients without intracorporeal suturing. Their technique involved the covering of the tumor bed and nephrectomy cavity layer-by-layer with FloSeal, Tisseel, and a fat pad after monopolar coagulation. More recently, Huang et al. [28] published a randomized controlled trial (RCT) to show that renal function was much better in the sutureless group of laparoscopic radiofrequency ablation-assisted tumor enucleation than the sutured group of conventional LPN. In our study, the eGFR we evaluated by serum creatinine changed little. We believe that the sutureless procedure preserves more renal function: this should be tested in future studies.

Many laparoscopic coagulators have been used for LPN, including the harmonic scalpel, the argon-beam coagulator, TissueLink, the microwave tissue coagulator, bipolar electrocautery, lasers, radiofrequency ablation, LigaSureTM, and others. The monopolar coagulator is the most common device used in electrical surgery. The coagulating modes of the monopolar coagulator include desiccate, fulgurate, and spray modes. We used the spray mode (100 W) and fulgurate mode (60 W) to coagulate the tumor bed, a new technique that to our knowledge has not been reported previously. Another advantage of the monopolar coagulation is its low cost. It did not incur any extra charges for materials or equipment.

NBCA is a monomer that polymerizes quickly, creating an acrylic resin that solidifies in less than 1 min [29]. NBCA has been used to achieve hemostasis and seal tissues in several surgical settings, including hernia repairs, lateral neck dissections, embolization procedures, and urethrocutaneous fistulae [30]. To the best of our knowledge, the present study is the first to report monopolar coagulation with NBCA in LPN. We found NBCA that helped form a helmet-like eschar that was hard enough to ensure hemostasis. We compared the patients with NBCA to ones without. The diameters, RENAL nephrometry scores, and item E of the RENAL scores of the tumors were significantly different. Three of the patients with NBCA had blood transfusions and two had positive surgical margins, because the tumors in the NBCA group were larger and more complex; however, there was no statistically significant difference between groups. For patients with exophytic (E item of the RENAL score = 1) and small (the diameter was less than 2 cm) renal tumors, NBCA was not necessary (Table 2).

**Table 2:** Outcomes of the NBCA group and the non-NBCA group

Characteristics	With NBCA	Without NBCA	*p* value
Patients (*n*)	98	44	
Age (years)	56 (20–86)	54.5 (34–78)	0.954
Gender			0.405
Male	62	31	
Female	36	13	
Diameters of tumor (cm)	3 (1.0–10.1)	2.0 (1.0–8.1)	< 0.001
RENAL nephrometry score	5.59	4.77	0.004
Low (4–6)	71	43	
Medium (7–9)	27	1	
E score			0.009
1	49	34	
2	49	10	
Operative time (min)	120 (50–200)	100 (40–180)	0.016
Mean estimated blood loss (ml)	100 (10–500)	50 (10–300)	0.101
Changes in eGFR 6 months (ml/min)	2.5	2	0.523
Complications (*n*)	3	0	0.241
Positive surgical margin (*n*)	2	0	0.34

**Fig. 3:**
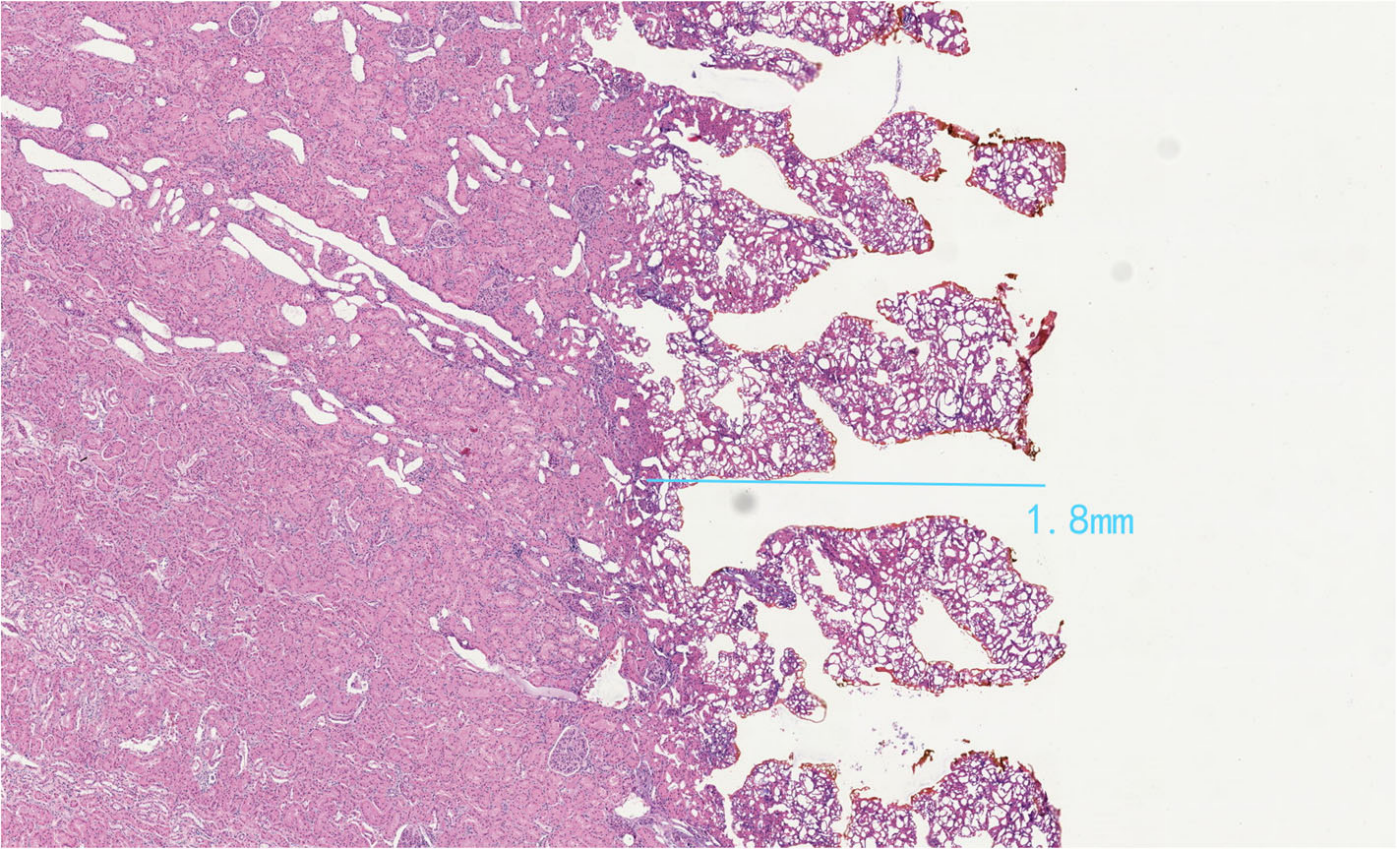
Microscopic details of damaged layer of a porcine kidney using monopolar coagulation (100 W spray mode and 60 W fulgurate mode)

We confirmed the damage depth created by monopolar coagulated using a porcine kidney. We used repeated monopolar coagulation in spray mode (100 W) and fulgurate mode (60 W) on a porcine kidney for 20 min. On microscopy, the damaged layer was only 1.8 mm deep (Fig. 3). Therefore, we believed that if the tumor was more than 2–3 mm above the collection system, the use of monopolar coagulation was safe. Nevertheless, to be on the safe side, we set 4 mm as the safe distance. Urine leakage did not occur after surgery.

This study was not without limitations. The total number of cases was limited. We did not have a prospective randomized controlled group, and we did not evaluate individual renal function by scintigraphy.

## Conclusions

Use of monopolar coagulation with NBCA in clampless and sutureless LPN for renal tumors with low RENAL nephrometry scores was safe and effective. For patients with exophytic renal tumors less than 2 cm, NBCA is not necessary.

## Abbreviations

CT: Computerized tomography
EBL: Estimated blood loss
eGFR: Estimated glomerular filtration rate
GFR: Glomerular filtration rate
LPN: Laparoscopic partial nephrectomy
NBCA: *N*-butyl-2-cyanoarcrylate
NSS: Nephron-sparing surgery
OPN: Open partial nephrectomy
PN: Partial nephrectomy
RCC: Renal cell carcinoma
RCT: Randomized controlled trial
RN: Radical nephrectomy
US: Ultrasound
WIT: Warm ischemia time
